# Remarks and Translations Illustrative of the Physiognomy of the Battle-field, and the Attitudes of the Dead

**Published:** 1860-07

**Authors:** Bennett Dowler


### REMARKS AND TRANSLATIONS,

### ILLUSTRATIVE OF THE PHYSIOGNOMY OF THE BATTLE-FIELD, AND THE ATTITUDES OF THE DEAD.

BY BENNETT DOWLER, M. D.

Like so many Alexanders,
Have, in these parts, from morn till even fought,
And sheathed their swords for lack of argument.

The poet, in every age, has labored to veil the hideousness of the battle-field, the aceldama where victory and glory are won. Here, scattering the flowers of song to the memory of the fallen, he invokes the noblest, mightiest, yet too often the most selfish passion, the love of country, right or wrong, as not only justifying carnage, but requiring unnumbered victims. It is sweet, it is honorable, says the Roman poet, to die for one's country:

"Dulce et decorum est pro patria mori."

"How sleep the brave, who sink to rest,
By all their country's wishes blest!"

But neither the masters of the world nor the poets can look the grim statistics of the killed and wounded in the face without comprehending their sad import, the unutterable anguish and bereavement of the families and friends of the slain and mutilated, and sympathising with the living and the dead.

The position of the medical man in the army, and particularly on the field of battle, though he is a non-combatant, is often one of danger, always one of usefulness, but never one of glory, in the military sense of that word. Amid dense smoke, the clangor of arms, the thunderings of artillery, and the rattling of shot, the surgeon, in modern wars, operates, often immediately on the battle-ground, where many of the wounded may be thus saved, who would otherwise perish in a few moments from hæmorrhage, etc. And when the battle is ended, his work at the hospital or ambulance still goes on, as amputations, disarticulations, trephinings, extracting bullets and other projectiles, dressing wounds, fractures, etc.; while the victorious combattants are partaking of the loud acclaims and blandishments of the public, enjoying festivities and amusements, honors, promotions and repose. The surgeon, in the meanwhile, hears, instead of the pæans of triumph, the groans of the mutilated, and the last sighs of the dying.

It appears that during the recent battles in Italy, some of the French physicians were directed by their superior medical officers, in addition to their more immediate duties to the living, to study the physiological mechanism, if one may so speak, of death itself, as it occurred on the battle-field—that is to say, the physiognomy, positions and attitudes incidental to death from the arms of war, during, or as soon as possible after, the conflict. Thus the surgeon passed from his operating ambulance to view the fallen. Is not this an intensification of the moral sublime? a unique study; original? French? more than tragedians ever conceived?

Thus Dr. Armand, physician-major of the first class, chief of the ambulance of the headquarters of the fourth corps of the French army of Italy, relates, from personal observation, some interesting particulars concerning the aspects and attitudes of the slain on the battle-fields of the Crimea and of Italy—a condensed translation or sketch of which (from the *Gaz. Hebdom. de Med.*, Sept. 16, 1859) will be subjoined, as worthy of consideration, psychically, physiologically, and traumatically.

During the day of the battle of Magenta, including the night, eight hundred wounded Frenchmen and Austrians underwent capital or minor operations and dressings at the ambulance of Dr. Armand. With his two assistants, he had completed his work by the dawn of the following day, when he proceeded to inspect the bloody field of Magenta, and the attitudes of the slain. A melancholy, not a useless study.

Dr. Armand observed that a great number of the dead preserved, as nearly as may be, the same attitudes in which they had been when the messengers of death struck them—a proof that they had passed from life to death without agony, without convulsions. Those struck in the head generally lay with the face and abdomen flat upon the ground, a position which the death-stiffness had not changed, holding, for the most part, their weapons still grasped in their hands.

Dr. Armand mentions a peculiarity often attendant upon wounds of the head, in which the patient thinks himself by no means dangerously hurt, although sometimes he dies, one might say, spontaneously, or by surprise. During the battle of Solferino, a soldier wounded in the head by a ball, entered the ambulance and was dressed by Dr. Lambert. The ball had perforated the skull and lodged in the cerebral mass; nevertheless, the patient's intelligence was perfect; he made light of his wound; lay down, having his lighted pipe in his mouth, with his head raised upon his knapsack against the wall, where he was found afterwards, with his pipe still in his mouth. He had expired without movement or noise. Dr. Armand details a similar case, that of a sergeant-major, whom Dr. Lambert (Dr. A.'s assistant) dressed in the Crimean war. The soldier smoked on for a dozen days after having been wounded; and having lighted his pipe for the last time, died suddenly, keeping it still in his mouth. These cases are, therefore, attested by at least two medical witnesses.

Dr. Armand says that soldiers who receive their death-wounds in the heart, fall and rest in the same manner as those who are killed by injury of the brain, though the death is not so instantaneous but that it may allow an attitude, which so to speak, is active. We have seen, among others, a Zouave struck fairly in the chest, who was brought together, or doubled upon his musket, as if taking a position to charge bayonet, his face full of energy, as if advancing with an attitude more menacing than that of a lion. It is reported that His Majesty had observed a similar case at Palestro.

On the other hand, an Austrian, who had died by hemorrhage from a ball which had divided the crural vessels, whose agony had been of some duration, as proven by the blood in which he was bathed, presented the attitude of supplication; he lay on his back, a little bent to the right, his face and eyes turned towards the heavens, both hands joined together, with the fingers interlaced and contracted. This man died in the attitude of prayer. In fact, religious ideas appeared to have prevailed quite extensively among the Austrian soldiers, as well as among the Russians in the campaign in the Crimea.

In the wounds of the abdomen, as the agony was more or less prolonged, the pains were intolerable, attended with vomiting and hiccough; the face of the corpse was was generally found contracted, the hands and forearms crossed and closed upon the abdomen, the body doubled upon itself, and resting on its side.

At Ponte Vecchio di Magenta, a hungarian hussar, killed, (as was his horse,) remained in the saddle, lying upon the right side, having the point of his sabre in advance, in the position of a horseman when charging. He had roses still fresh in his tolpak, his forehead pierced with a ball; his horse was riddled with shot in the head, and both had died simultaneously. This case was witnessed by Dr. A. Renanrd. Dr. Armand relates a parallel case which occurred to an Austrian artilleryman.

At Melegano, several French soldiers, while charging bayonet, fell mortally wounded with grape-shot; their faces rested on the ground, and their bayonets pointed in advance.

At Magenta among the slain strewed upon the battle-ground several Austrian officers were recognized of distinguished physiognomy, dressed with the utmost care and propriety in glossy gloves—one might say that they had affectedly made their toilette in anticipation of death. Their fine blonde heads of hair and regular features, for the most part different from the common soldiers, had the expression of bravery and resignation.

Next to such a vast panorama of death, the dead-house of the Charity Hospital of New Orleans, during the great epidemic of yellow fever, may claim a place. The physiognomy of the yellow fever corpse is usually sad, sullen, and pertubed; the countenance dark, mottled, yellow, livid and bloodshotten. The veins of the face, and of the whole body, often become distended; the whole expression is less calm and placid than in most other corpses, especially such as have died of hæmorrhages.

The following tableau of the dead body of Goethe is by the masterly hand of Eckerman:

"The morning after Goethe's death, a deep longing seized me to look yet once again upon his earthly garment. His faithful servant, Frederic, opened for me the chamber in which he was laid out. Stretched upon his back, he reposed as if in sleep; profound peace and serenity reigned in the features of his noble, dignified countenance; the mighty brow seemed yet the dwelling-place of thought. I wished for a lock of his hair, but reverence prevented me from cutting it off. The body lay naked, only wrapped in a white sheet; large pieces of ice had been placed around, to keep it fresh as long as possible. Frederic drew aside the sheet, and I was astonished at the divine magnificence of the form. The breast was so powerful, broad, and arched; the limbs full and softly muscular; the feet elegant, and of the most perfect shape; nowhere on the whole body a trace either of fat or of leanness and decay; a perfect man lay in great beauty before me; and the rapture which the sight caused, made me forget for a moment, that the immortal spirit had left such an abode. I laid my hand on his heart—there was a deep silence—and I turned away to give free vent to my tears."

Independent of experience, the physiologist cognizes no inherent necessity in life itself, nor in any of its organized forms of manifestation, nor in any of its structural adaptations and finalities, for a catastrophe so melancholy—so repugnant to the instincts of humanity as death. Indeed, the analogies of the material universe wherein stability reigns, or varies only in constantly recurring cycles, seem to teach that man, for whom all things appear to exist, is, what his irrepressible instincts claim, immortal—exempt from death! The stars rise undiminished as on the morning of creation, and "pursue the even tenor of their way" through infinite space. The earth, a little scarred on its face by volcanic eruptions and accidents, undecayed by age, "spins silently onward with spheres which never sleep; her unwithered countenance being as bright as at creation's day." Trees live thousands of years and some fishes for centuries. The inferior animals neither foreknow nor apprehend impending death at every step in life. This unpleasant secret is made known to man alone. A current, he can no more resist than the unfortunate boatman caught by the descending rapids of Niagara, hurries him over a precipice into a realm as tenebrious (after all the researches of mere physical science) as that into which the fabled Styx debouched in the days of antiquity.

Poets and philosophers have sought to bring out in the foreground pictures more cheering, so as to veil the sombre tableau of death in the distance. Bryant's picture is one of the most pleasing:

"So live, that, when thy summons comes to join
The innumerable caravan that moves
To the pale realms of shade, where each shall take
His chamber in the silent halls of death,
Thou go not like the quarry slave at night,
Scourged to his dungeon: but, sustained and soothed
By an enfaltering trust, approach thy grave
Like one who wraps the drapery of his couch
About him, and lies down to pleasant dreams."

With unsurpassed beauty, La Fontaine calls death the evening of a fine day:

"La mort est le soir d'un beau jour." (*N. O. Med. and Surg. Jour.*

